# Vascular Endothelial Barrier in Salivary Glands: From Physiological Regulation to Pathological Impairment of Secretion

**DOI:** 10.3390/ijms27136076

**Published:** 2026-07-07

**Authors:** Sai-Nan Min, Li-Ling Wu, Guang-Yan Yu, Xin Cong

**Affiliations:** 1 First Clinical Division, Peking University School and Hospital of Stomatology & National Center of Stomatology & National Clinical Research Center for Oral Diseases & National Engineering Research Center of Oral Biomaterials and Digital Medical Devices, Beijing 100034, China; sainanmin2019@bjmu.edu.cn; 2 Department of Physiology and Pathophysiology, Peking University School of Basic Medical Sciences, State Key Laboratory of Vascular Homeostasis and Remodeling, Beijing 100191, China; pathophy@bjmu.edu.cn; 3 Department of Oral and Maxillofacial Surgery, Peking University School and Hospital of Stomatology & National Center of Stomatology & National Clinical Research Center for Oral Diseases & National Engineering Research Center of Oral Biomaterials and Digital Medical Devices, Beijing 100081, China; gyyu@263.net

**Keywords:** salivary gland, endothelial barrier, tight junction, claudin-5, hyposalivation

## Abstract

Although salivary glands are highly vascularized, the microvascular endothelial barrier has only recently emerged as a pivotal determinant of glandular homeostasis and disease. This review synthesizes current understanding of the salivary gland endothelial barrier, with particular emphasis on the regulation of tight junctions (TJs). Structurally, the barrier comprises endothelial cells interconnected by TJs and adherens junctions, supported by a basement membrane and pericytes. Among TJ components, claudin-5 serves as a key endothelial-specific regulator of paracellular permeability, and is dynamically modulated by biochemical and mechanical stimuli during saliva secretion. Cholinergic, adrenergic, and neuropeptide signaling pathways coordinate to fine-tune endothelial permeability to meet the fluctuating secretory demands. Conversely, under pathological conditions, such as Sjögren’s syndrome, radiation-induced injury, diabetes mellitus, fibrotic diseases, and salivary gland tumors, the integrity of the endothelial TJ complex is impaired. These pathologies are characterized by aberrant TJ expression, mislocalization, and signaling-mediated junctional disassembly, which trigger vascular leakage and immune cell infiltration—two key processes that act as primary drivers of glandular dysfunction. Collectively, these findings enrich our understanding of the microvascular mechanisms that link endothelial barrier function to salivation, and highlight that the restoration of junctional integrity is a promising therapeutic strategy for salivary gland diseases.

## 1. Introduction

Saliva is a multifunctional biological fluid that is essential for oral lubrication, digestion, mucosal immunity, microbial homeostasis, and wound healing [1,2]. Hyposalivation has been identified as a marker closely associated with increased all-cause mortality among community-dwelling Japanese men aged 70 years and older [3]. While conventional models of saliva production primarily emphasize acinar cell secretion and subsequent ductal modification, the microvascular exchange between the systemic circulation and the glandular interstitium remains an underappreciated determinant of the ultimate salivary composition.

Capillaries within major salivary glands form dense networks that intimately surround acini and ducts, thereby optimizing nutrient delivery and fluid exchange. The vascular endothelium establishes a barrier that not only separates the circulating blood compartment from the glandular interstitium but also actively prevents paracellular leakage and selectively regulates the passage of molecules through complex intercellular junctions, including tight junctions (TJs) and adherens junctions (AJs) [4]. Therefore, the integrity and permeability of the endothelial barrier exert a critical influence on both the quantitative and qualitative features of saliva [5]. Transcriptomic analyses further underscore the specialized identity of these endothelial cells, revealing transcriptional profiles that cluster closely with those of colon and a subset of small intestinal endothelial cells, alongside the expression of more than a thousand unique genes absent in other organ-specific endothelial populations [6]. Despite this profound structural and molecular specialization, the vascular endothelial barrier has only recently emerged as a key player in salivary gland physiology and pathology.

Endothelial dysfunction is increasingly recognized as a central driver of salivary gland pathologies characterized by hyposalivation and altered saliva composition, including autoimmune sialadenitis such as Sjögren’s syndrome (SS), radiation-induced injury, diabetes, fibrotic diseases, and salivary gland tumors [7,8,9,10,11]. This review outlines the physiological architecture and functional dynamics of the salivary endothelial barrier, with a specific focus on the regulation of TJs. Furthermore, we delineate the pathogenic mechanisms underlying endothelial barrier breakdown in inflammatory and fibrotic diseases of the salivary glands, and highlight the therapeutic strategies targeting endothelial barrier.

## 2. Physiological Architecture of the Salivary Gland Endothelial Barrier

**Figure 1:**
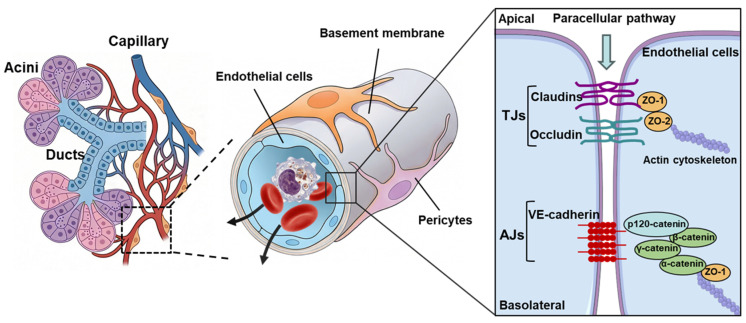
Scheme of physiological architecture of the salivary gland endothelial barrier. AJ, adherens junction; TJ, tight junction; ZO, zonula occludens.

The organization and components of the endothelial barrier vary significantly across vascular beds [12]. Unlike blood–brain barrier capillaries that exhibit extremely high trans-endothelial electrical resistance exceeding 1500 Ω·cm^2^ [13], salivary gland capillaries maintain a more permeable barrier to accommodate high rates of plasma protein filtration and immune cell extravasation [14]. This fine-tuned permeability reflects the dual function of gland, accommodating high rates of plasma protein filtration and fluid exudation to sustain saliva secretion while enabling controlled immune surveillance to detect pathogens without excessive leakage. Structurally, this specialized barrier comprises three integrated components (1) a continuous endothelial monolayer sealed by TJs and AJs; (2) an underlying basement membrane rich in collagen IV and laminin; and (3) a surrounding network of pericytes [15] (Figure 1). Together, these components dynamically integrate biochemical cues and mechanical forces to govern transvascular transport.

### 2.1. Endothelial TJ Structure

In neighboring endothelial cells, TJs are junctional complexes that regulate the paracellular passage of materials across the polarized monolayer [12]. TJs also maintain cell polarity by acting as a fence, preventing the free diffusion of lipids and proteins between apical and basolateral membranes [16]. TJs are composed of over 40 distinct proteins, primarily classified into transmembrane proteins, such as claudins and occludin, and cytosolic scaffolding proteins, such as zonula occludens (ZOs). The claudin family consists of 20–27 kDa tetraspan transmembrane proteins characterized by two extracellular loops and cytosolic N- and C-termini. Through these extracellular loops, claudins on adjacent epithelial or endothelial cells engage in intercellular interactions, creating a paracellular “zipper”. This zipper-like assembly rigorously governs the tightness of the TJ barrier and determines paracellular permeability based on molecular size and charge selectivity [17]. To date, 27 claudin family members have been discovered in mammals [18]. They form TJ structures that act either as barriers against ions and solutes such as claudin-1, -3, -5, -8, -11, -18, and -19 [19,20,21,22,23,24,25], or as selective paracellular ion pores such as claudin-2, -4, -10, -15, -16, -17, and -21 [26,27,28,29,30,31,32]. Intriguingly, claudins in endothelial cells tend to be barrier-forming molecules. Among them, claudin-5 represents the most highly enriched, endothelium-specific sealing component within vascular endothelial cells [21]. The indispensable role of claudin-5 is demonstrated at the blood–brain barrier, where it strictly restricts the paracellular passage of molecules smaller than 800 Da. Claudin-5-deficient mice exhibit size-selective leakage of the blood–brain barrier, resulting in early postnatal lethality despite macroscopically normal blood vessel formation [33]. Collectively, claudins serve as the fundamental backbone of TJs, with endothelium-enriched members like claudin-5 acting as the indispensable gatekeepers of vascular barrier integrity.

As the first identified tetraspan transmembrane TJ protein, occludin integrates into claudin-based TJ strands to seal the intercellular space and maintain vascular barrier integrity [34]. Structurally, it comprises two tyrosine- and glycine-rich extracellular loops, a short N-terminal domain, and a long C-terminal cytoplasmic domain. Interestingly, while intact TJ structures can still form in occludin-null mice, suggesting it may not be strictly essential for initial baseline assembly, occludin remains indispensable for complex tissue homeostasis [35]. This is evidenced by the diverse phenotypic abnormalities observed in mice lacking occludin, including brain vessel calcification, postnatal growth retardation, thinning of compact bone, salivary gland defects, gastric inflammation, and testicular atrophy, all of which point to multifaceted functions of occludin that warrant further investigation [35]. Far from being a mere static structural wall, occludin functions as a highly dynamic signaling hub. Its two extracellular loops are subject to extensive post-translational modifications, particularly phosphorylation, which orchestrate its assembly within the TJ complex and dictate junctional stability [36,37,38]. Furthermore, occludin actively participates in intracellular signaling cascades that modulate paracellular permeability. Under pathological conditions, inflammatory cytokines such as tumor necrosis factor α and other stimuli trigger signaling cascades like the p38 mitogen-activated protein kinase (MAPK) pathway, the Ras homolog gene family member A (RhoA)/Rho-associated coiled-coil kinase (ROCK) pathway, and Src family kinases signaling. Alteration in these pathways induces the aberrant phosphorylation or dysregulation of occludin, leading to junction disassembly, cytoskeletal remodeling, and heightened vascular leakage [12]. Taken together, occludin links structural integrity with intracellular signaling to dynamically control endothelial permeability.

Transmembrane TJ proteins are anchored to the cytoplasmic scaffold via highly conserved C-terminal PDZ-binding motifs, which interact with the ZO protein family (ZO-1, ~220 kDa; ZO-2, ~160 kDa; and ZO-3, ~130 kDa) [39]. The ZO family acts as cytoplasmic scaffold proteins that stabilize the TJ complex by crosslinking transmembrane proteins to the actin cytoskeleton and various signaling molecules, thereby enabling the dynamic regulation of junctional integrity [40]. In addition to the classical claudin–ZO axis, numerous transmembrane receptors that interact with ZO proteins, particularly immunoglobulin-like superfamily adhesion molecules including the coxsackievirus and adenovirus receptor (CAR). Functionally, CAR acts as a transmembrane adhesion molecule governing intercellular adhesion attachment and barrier integrity. Mechanistically, it directly binds to scaffolding proteins containing multiple PDZ domains, particularly ZOs and multi-PDZ domain protein 1 (MUPP1), thereby anchoring the transmembrane adhesion complex to the underlying actin cytoskeleton [41]. Functional cellular evidence demonstrated that CAR overexpression suppresses transepithelial passage of ions and macromolecules, whereas soluble CAR disrupt TJ formation [42]. Genetic knockout models further showed the physiological significance of CAR: adult CAR-knockout mice exhibit severe, widespread multi-organ defects, encompassing intestinal dilation, exocrine pancreatic atrophy accompanied by acinar-to-ductal metaplasia, impaired electrical signal propagation associated with structural defects between myocytes, and abnormal thymopoiesis [43]. The already known function of CAR in epithelia as a tight junction protein together with its biological functions have been documented in a previous review [41]. Taken together, the ability of ZO-1 to coordinate interactions with diverse transmembrane proteins, including claudins and immunoglobulin-like superfamily members such as CAR, highlights the critical role of ZO-1 in the assembly of TJ complexes and in maintaining both epithelial and endothelial homeostasis.

The expression profiles and specific barrier functions of TJ proteins exhibit significant tissue and cellular heterogeneity. In recent years, studies on the significance of TJ proteins in salivary glands have predominantly focused on epithelium across various species [5]. However, recent evidence highlights the well-organized, continuous TJ networks within the salivary gland capillary endothelium. Morphologically, under physiological conditions, the endothelial TJ width in the mouse submandibular gland is larger than that of acinar and ductal cells, suggesting a higher permeability for microvascular fluid exchange [44]. The molecular composition of endothelial TJ complex is distinctly segregated from that of the glandular epithelium. Immunohistochemical analyses in both human and rodent major salivary glands reveal that claudin-5 is strictly confined to microvascular endothelial cells [45,46,47]. Claudin-1, while present in microvessels, is also shared with subsets of ductal cells, whereas other claudins (e.g., claudin-3 and -4) are strictly restricted to epithelial compartments [45]. Meanwhile, occludin and ZO-1 are ubiquitously distributed across acini, ducts, and endothelia, forming continuous belts at cell–cell contacts [45], while their specific regulatory mechanisms in maintaining salivary vascular homeostasis remain poorly characterized. Furthermore, the assembly of the TJ complex is highly dynamic during organogenesis. Developmental studies document temporal and spatial shifts in multiple claudins including claudins-3 to -8, -10, and -11, during salivary gland branching morphogenesis, with robust claudin-5 signals persistently identifying the assembling vascular networks within the mesenchyme [47,48]. Together, these findings establish claudin-5 as a vascular endothelial marker in salivary tissue and underscore the distinct molecular architectures that differentiate endothelial from epithelial TJs.

### 2.2. Endothelial AJ Structure

While TJs govern paracellular permeability, AJs provide the fundamental mechanical stability required for barrier integrity. In the vascular endothelium, AJs are primarily organized by vascular endothelial-cadherin (VE-cadherin), an endothelium-specific adhesion molecule that mediates Ca^2+^-dependent homophilic interactions between adjacent cells [49]. To establish structural stability, the cytoplasmic domain of VE-cadherin anchors to the actin cytoskeleton via catenin family proteins. Within this complex, p120-catenin binds to the juxtamembrane domain to prevent VE-cadherin internalization, whereas β-catenin and γ-catenin interact with the C-terminal regions to anchor α-catenin [50,51]. Endothelial permeability is dynamically regulated through the remodeling of the VE-cadherin–catenin complex and associated cytoskeletal structures. For example, inflammatory mediators and growth factors can trigger the phosphorylation of VE-cadherin or its associated proteins, driving catenin dissociation, VE-cadherin internalization, and subsequent barrier leakiness [52,53,54]. Furthermore, AJs do not act in isolation but engage in profound spatiotemporal crosstalk with TJs to cooperatively regulate the endothelial barrier [55]. The TJ scaffold ZO-1 actively modulates the structural integrity and mechanical tension of AJs by recruiting Rho-GTPases signaling molecules such as p114-RhoGEF, thereby stimulating local actomyosin activation and physically coupling the core VE-cadherin complex to the cellular force-generating machinery [56]. Increasing evidence also indicates that VE-cadherin interacts with multiple signaling receptors, including vascular endothelial growth factor (VEGF) receptors, and participates in mechanosensory complexes that respond to fluid shear stress, highlighting its dual structural and signaling roles in endothelial biology [57,58,59]. These studies demonstrate that endothelial AJs function as highly dynamic platforms that integrate mechanical cues and signaling pathways to control vascular permeability and leukocyte extravasation. Although AJs are ubiquitously distributed and well-studied in endothelial cells, their specific structural configuration and functional contribution to the endothelial barrier within the salivary gland remain largely elusive.

### 2.3. Basement Membrane and Pericyte Support

The structural integrity of the capillary bed is also dependent on the subendothelial basement membrane and pericyte coverage. The basement membrane is a thin (~100 nm) meshwork comprising type IV collagen, laminin, perlecan, and nidogen, which provides structural support and biochemical signaling. Abluminal to the endothelial layer, pericytes establish a tight structural alliance with the microvasculature [15]. In both murine and human salivary glands, pericytes are typically identified by the expression of platelet-derived growth factor receptor β (PDGFRβ) and a distinct elongated morphology with cytoplasmic processes extending along capillaries [60]. Although they constitute a relatively minor cellular subset, pericytes maintain a highly consistent presence within glandular tissues, comprising 2.9–3.8% of the total cell population in mice and approximately 3.1% in human parotid and submandibular glands [60]. This strategic perivascular localization allows pericytes to play pivotal roles in maintaining vascular stability, microcirculation, and the stem cell niche within the gland. Consequently, the disruption of this supportive microenvironment has profound pathological implications. Loss of pericyte coverage or basement membrane damage, which occurs in SS and other pathological states, predisposes endothelial cells to junctional disassembly, leading to exacerbated vascular permeability and immune cell infiltration [15,60].

## 3. Physiological Regulation of the Endothelial Barrier in Salivary Secretion

**Figure 2:**
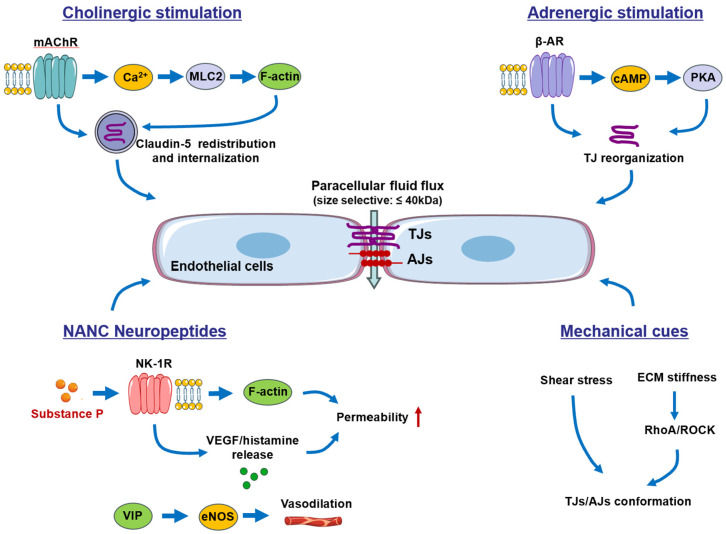
Physiological regulatory mechanism of endothelial cell junctions during saliva secretion under distinct secretory stimulation. AJ, adherens junction; cAMP, cyclic AMP; ECM, extracellular matrix; eNOS, endothelial nitric oxide synthase; mAChR, muscarinic acetylcholine receptor; MLC, myosin light chain; NANC, non-adrenergic, non-cholinergic; NK-1R, neurokinin-1 receptor; PKA, protein kinase A; TJ, tight junction; VEGF, vascular endothelial growth factor; VIP, vasoactive intestinal peptide; β-AR, β-adrenergic receptor.

The regulation of the salivary microvascular endothelium is a highly sophisticated, stimulus-responsive process. Table 1 summarizes the normal versus pathological control of the endothelial barrier in the salivary gland. Under physiological conditions, the robust generation of primary saliva depends crucially on the continuous and precisely controlled extravasation of plasma fluid from the local microcirculation [61]. This trans-endothelial fluid flux is strictly governed by endothelial junctional complexes, predominantly TJs such as claudin-5. Unlike epithelial barriers, salivary endothelial TJs exhibit profound plasticity, allowing them to rapidly adapt to varying secretory demands. Below, we provide a detailed review of the dynamic alterations and regulatory mechanisms governing these endothelial cell junctions during saliva secretion under distinct secretory stimulation (Figure 2).

### 3.1. Cholinergic (Parasympathetic) Stimulation

Parasympathetic innervation, acting primarily through acetylcholine and its pharmacological analogs including pilocarpine and carbachol, orchestrates the voluminous, fluid-rich phase of saliva secretion [62]. Our in vivo studies have provided direct visual evidence that cholinergic stimulation profoundly and rapidly remodels the salivary microvascular endothelial barrier [44]. Under resting conditions, the salivary microvasculature exhibits a baseline permeability strictly limited to small molecules (≤4 kDa), remaining completely impermeable to larger macromolecules [44]. However, upon pilocarpine-induced muscarinic acetylcholine receptor (mAChR) activation, the endothelial barrier transiently opens. Real-time two-photon imaging demonstrates an accelerated paracellular flux of both 4 kDa and 40 kDa macromolecules into the basal interstitium of the acini [44]. Crucially, this fenestration is size-selective, and the barrier remains impermeable to large serum proteins such as 70-kDa dextran and albumin, thereby preventing pathological tissue edema while ensuring an adequate fluid supply for transepithelial transport. Mechanistically, the transient hyperpermeability is driven by the rapid remodeling of claudin-5, the signature TJ protein of vascular endothelium. Cholinergic stimulation triggers the displacement of claudin-5 from apicolateral junctional complexes, prompting its lateral and basolateral redistribution, as well as partial cytoplasmic internalization [44]. This dynamic reorganization is primarily regulated by the myosin light chain 2 (MLC2)–F-actin signaling axis. mAChR activation triggers a rapid intracellular Ca^2+^ transient that leads to the phosphorylation of MLC2, which subsequently induces actomyosin contractility and rearrangement of the peri-junctional F-actin cytoskeleton [44]. Because endothelial TJs are deeply anchored to the actin cytoskeleton through scaffolding proteins, this actomyosin contraction generates mechanical tension that destabilizes the claudin-5–based junctional strands, facilitating rapid paracellular fluid flux required for salivary secretion.

### 3.2. Adrenergic (Sympathetic) Stimulation

While the cholinergic system primarily drives the rapid mobilization of fluid during salivary secretion, sympathetic stimulation, mediated by noradrenaline or β-adrenergic agonists, predominantly drives the exocytosis of pre-formed secretory granules from acinar cells, generating a more viscous, protein-rich saliva [62,63]. The reduced demand for large-scale trans-endothelial fluid flux implies a distinct adaptive response from the salivary microvascular endothelium, yet its direct response to β-adrenergic signaling remains poorly defined.

In salivary gland epithelial cells, established evidence indicates that β-adrenergic signaling engages a cyclic AMP (cAMP)/canonical protein kinase A (PKA)-dependent pathway that rapidly tunes secretion. For example, isoproterenol induces a rapid and transient phosphorylation of the water channel aquaporin 5 at Thr259 through cAMP/PKA in submandibular and parotid gland cells in vivo [64]. Studies using ultrastructural tracers demonstrate that isoproterenol temporally increases tight junctional permeability to myoglobin (17.8 kDa), whereas α1-adrenergic stimulation does not similarly increase permeability [65]. Consistent with a chronic remodeling phenotype, long-term repeated isoproterenol treatment also induces pronounced tight-junction structural reorganization in submandibular acinar cells, including increased tight-junction strand number and altered apical–basal depth, reinforcing the notion of adrenergically driven barrier plasticity within salivary gland epithelial compartments [66].

Although direct experimental evidence for adrenergic stimulus-specific TJ remodeling within the salivary microvasculature remains limited, insights from general vascular biology reveal that PKA plays highly diverse, context-dependent roles across different endothelial models, participating in both the assembly and disassembly of TJs [67,68,69,70,71,72,73]. Whether β-adrenergic signaling also orchestrates a size-selective TJ opening in the salivary microvasculature, and how this dynamic loosening coordinates with glandular exocytosis, remains an important question that warrants further investigation.

### 3.3. Non-Adrenergic, Non-Cholinergic (NANC) Neuropeptides

During intense reflex-mediated salivation, autonomic nerves co-release NANC neuropeptides, most prominently substance P (SP) and vasoactive intestinal peptide (VIP) [74,75]. These potent vasoactive mediators cooperate with classical neurotransmitters to fine-tune vascular responses within the salivary gland microenvironment. SP, binding to neurokinin-1 receptors, is a potent stimulant of salivary secretion. In rat salivary glands, intra-arterial infusion of SP leads to dose-dependent increases in both parotid and submandibular gland secretion [76]. A substance P antagonist inhibits the entry of horseradish peroxidase (40 kDa) into saliva through TJs in rat submandibular glands induced by 20 Hz stimulation of the chorda tympani nerve [77]. SP also plays a trophic role, as continuous infusion of SP can prevent the weight loss and atrophy of parotid glands caused by parasympathetic denervation or dietary changes, suggesting its importance in maintaining gland health [78]. Several studies have shown that SP can increase endothelial permeability in other types of endothelial cells, likely through the induction of stress fibers, redistribution of F-actin, and the secretion of VEGF, rather than directly via accelerated redistribution and internalization of claudin-5-containing TJ complexes [79,80,81]. However, the role of SP in the modulation of endothelial barrier in salivary glands has not been extensively investigated. In the salivary gland, SP has been reported to enhance vascular permeability by indirectly releasing histamine, leading to increased plasma extravasation [82]. Moreover, alterations in SP levels and neurokinin-1 receptor expression have been observed in minor salivary glands of patients with primary SS, correlating with impaired secretion, highlighting its potential involvement in salivary dysfunction [83]. In contrast, VIP primarily regulates vascular tone rather than directly promoting junctional disassembly. Through activation of endothelial nitric oxide synthase (NOS) and generation of nitric oxide (NO), VIP induces pronounced local vasodilation within the salivary gland microcirculation, generating an adequate fluid reservoir for secretion [84,85]. Together, these NANC neuropeptides act as complementary modulators of the salivary microvasculature: SP actively enhances endothelial permeability, whereas VIP orchestrates hemodynamic conditions favorable for secretion.

### 3.4. Mechanical Cues and Hemodynamic Forces During Secretion

Although biochemical signals initiate salivary secretion, the secretory process itself drastically alters the physical and hemodynamic landscape of the glandular microvasculature. Autonomic stimulation triggers massive surges in local blood flow, generating acute luminal shear stress. Simultaneously, the active contraction of myoepithelial cells physically compresses the acini, exerting mechanical strain on adjacent capillary beds, while the rapid extravasation of paracellular fluid acutely elevates interstitial pressure. Furthermore, pathological extracellular matrix stiffness is a hallmark of various salivary gland diseases such as SS and salivary adenoid cystic carcinoma [86,87]. Evidence suggests that salivary gland epithelial cells actively sense extracellular matrix mechanical cues. For instance, isolated mouse embryonic submandibular glands cultured on soft substrate (0.48 kPa) exhibit normal morphogenesis and differentiation, whereas culture on a stiff substrate (19.66 kPa) significantly impairs branching morphogenesis and disrupts aquaporin 5 expression. Notably, transferring glands from 19.66 to 0.48 kPa gels restores acinar structure and differentiation [88]. Substrate stiffness also regulates the migratory and invasive ability of salivary adenoid cystic carcinoma cells via RhoA/ROCK pathway [87]. These biomechanical forces are not confined to the epithelium but also critically regulate endothelial junctional integrity. Biomechanical forces, including fluid shear stress, extracellular matrix stiffness, and cyclic strain, are known to critically regulate the conformation of TJs and AJs, thereby governing vascular permeability [89,90]. For example, under physiological shear stress levels, endothelium typically adopts a more mature phenotype characterized by a linear distribution of VE-cadherin at cell–cell borders and perimeter-directed actin filament rearrangement [91]. Moreover, endothelial mechanosensitivity to extracellular matrix stiffness exhibits remarkable tissue-specific heterogeneity [92]. However, direct studies mapping these biomechanical forces within the salivary microcirculation are currently lacking. Elucidating how intersecting mechanical cues drive dynamic spatiotemporal remodeling of endothelial junctions during both physiological salivation and pathological gland dysfunction is a critical future direction.

**Table 1:** Normal versus pathological control of the endothelial barrier in salivary glands.

Category	Stimulus	Junctional Targets	Regulatory Routes	Permeability Consequence	Level of Proof
Physiological control (normal)	Cholinergic (e.g., pilocarpine)	TJs (e.g., claudin-5)	Transient, reversible TJ opening to facilitate fluid transport during secretion	Transient “leakage” for function	In vivo mouse study [44]
	Adrenergic (e.g., isoproterenol)	NA	Increases tight junctional permeability, and induces TJ reorganization	Increases permeability to myoglobin	In vivo rat study [65]
	NANC neuropeptides (e.g., substance P and VIP)	NA	Releases histamine, induces vasodilation, and increases blood flow	Indirectly affects permeability	In vivo rat/dog/cat studies [82,84,85]
Pathological disruption (disease)	Inflammatory cytokines (e.g., IFN-γ and MCP-1)	Claudin-5	Upregulates claudin-5 levels, induces mislocalization from junction to cytoplasm, and activation of ERK1/2 signaling pathway	Increases permeability, facilitates lymphocyte infiltration, and drives fibroblast activation	In vivo (NOD mouse; murine ligation model), in vitro (HUVEC), human tissue (SS, IgG4-RS) [7,10,93]
	Ionizing radiation (e.g., 10–15 Gy)	TJs (e.g., occludin, claudin-5, and ZO-1), AJs (e.g., VE-cadherin), and pericytes	Reduces mRNA expression of junctional proteins, and causes apoptosis	Induces microvessel loss and pericyte depletion	In vivo mouse studies [8,60,94]
	Hyperglycemia	TJs (e.g., occludin and ZO-1)	Reduces expression of junctional proteins	Increases permeability, and contributes to hyposalivation	In vivo (db/db mouse), in vitro (HUVEC) [95,96]
	Tumor-derived Factors (e.g., VEGF)	VE-cadherin, occludin	Pro-angiogenic signaling (VEGF-R)	Increases permeability, induces abnormal angiogenesis, and facilitates intravasation	Human tissue analysis, scRNA-seq [97,98,99]

AJ, adherens junction; HUVEC, human umbilical vein endothelial cell; IFN-γ, interferon-γ; IgG4-RS, immunoglobulin G4-related sialadenitis; MCP-1, monocyte chemoattractant protein-1; NA, not available; NANC, non-adrenergic, non-cholinergic; NOD, non-obese diabetic; SS, Sjögren’s syndrome; TJ, tight junction; VEGF, vascular endothelial growth factor; VIP, vasoactive intestinal peptide.

## 4. Vascular Barrier Disruption in Salivary Gland Diseases

**Figure 3:**
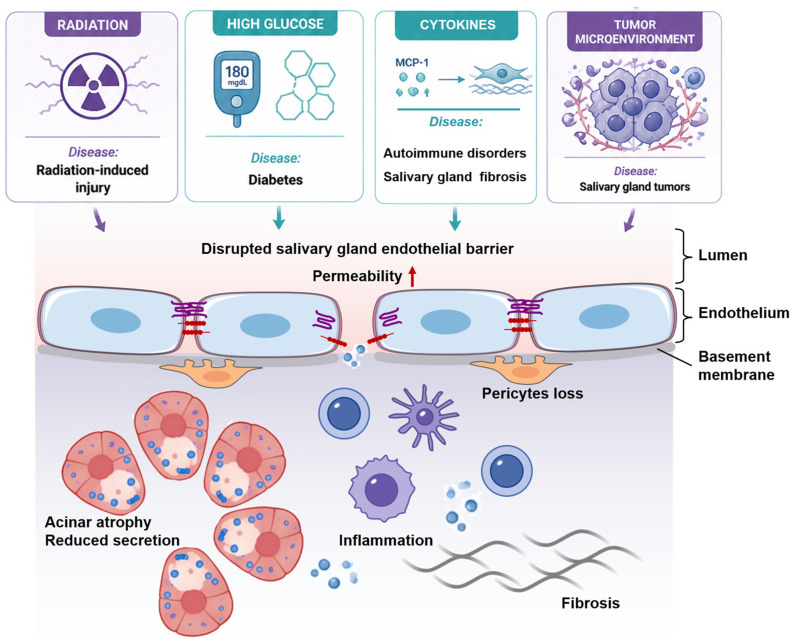
Vascular barrier disruption in salivary gland diseases under pathological conditions.

Endothelial TJs are highly responsive to microenvironmental changes and play a critical role in maintaining vascular homeostasis. Various pathological stimuli, including inflammatory cytokines, radiation, and metabolic stress, can compromise the endothelial barrier through the altered expression, mislocalization, or post-translational modification of TJ and AJ proteins, or the loss of pericytes [4,12]. Once cell junction architecture is disrupted, the resulting vascular leakage of fluids, macromolecules, and immune cells profoundly impairs the function of surrounding tissues. Emerging evidence considers vascular barrier disruption not merely as a secondary symptom in salivary glands, but as a primary pathogenic driver across a wide spectrum of secretory disorders. The following sections detail how microvascular damage and endothelial barrier disruption contribute to the progression of these distinct salivary gland pathologies, highlighting the restoration of endothelial barrier integrity as a promising therapeutic target (as detailed in Table 2 and Figure 3).

### 4.1. Autoimmune Disorders

SS is a chronic inflammatory autoimmune disorder characterized by progressive perivascular infiltration of lymphocytes and destruction of exocrine glands, including salivary and lacrimal glands, which results in xerostomia and xerophthalmia. While the classical pathogenic models focus on epithelial damage and acinar loss [100], mounting evidence indicates that vascular endothelial barrier disruption is a primary mechanism that enables excessive lymphocyte infiltration and amplifies local autoimmune responses. Clinically, the flow-mediated dilation value of the brachial artery in SS patients is significantly lower than in controls, indicating systemic endothelial dysfunction [101]. In non-obese diabetic (NOD) mouse model for SS, diameters of microvessels and blood flow rate are higher than those of Balb/c mice, and the endothelial TJ width is increased [7]. The leakage of 4, 40, 70 kDa fluorescein isothiocyanate (FITC)-dextran from vessels to the basal sides around acini has been observed in the unstimulated submandibular glands of 12- and 21-week-old NOD mice. This leakage is accompanied by a decreased response to pilocarpine, which is responsible for the hyposalivation. In the salivary glands of both SS patients and NOD mice, the expression of claudin-5 is upregulated and redistributed from apicolateral membrane into cytoplasm, resulting in a defective endothelial barrier. The redistribution is considered a crucial pathogenic factor facilitating inflammatory cells infiltration into glandular lesions [7]. In a cultured human microvessel endothelial cell line, the stimulation of interferon-γ (IFN-γ), a cytokine elevated in SS, increases the mRNA and protein levels of claudin-5, implicating IFN-γ as a critical pathogenic mediator of endothelial TJ disruption in SS [7].

Immunoglobulin G4-related sialadenitis (IgG4-RS) is another autoimmune fibro-inflammatory disease involving salivary glands, pathologically characterized by extensive IgG4^+^ plasma cells infiltration and storiform fibrosis. Our previous proteomics analysis in IgG4-RS identified 272 upregulated and 127 downregulated expressed protein, which are enriched in pathways related to leukocyte transendothelial migration and saliva secretion, suggesting that endothelial barrier disruption might be involved in the pathogenesis of IgG4-RS [93]. Specifically, the mRNA expression of claudins including claudin-3, -4, -6, -7, -8, -10, and -12, occludin, and ZO-1 are reduced in the submandibular glands of IgG4-RS patients. Conversely, the endothelium-specific sealing protein claudin-5 is elevated and mislocalized from the apicolateral membrane to the lateral membrane and cytoplasm, a pattern consistent with that seen in SS [10,93]. The enhanced intensity of phosphorylated extracellular signal-regulated kinase 1/2 (p-ERK1/2) in the nucleus of endothelial cells suggests that p-ERK1/2 pathway may serve as the primary signaling cascade driving this claudin-5 redistribution [10].

### 4.2. Radiation-Induced Salivary Gland Dysfunction

Radiation therapy for head and neck cancer damages salivary glands not only through direct injury to acini, but also through vascular damage, ultimately resulting in severe hyposalivation. Demonstrating this vascular vulnerability, microvessel density in murine salivary glands decreases by 45% within just four hours of a 15 Gy radiation dose [8]. However, pretreating mice with a serotype 5 adenovirus vector encoding either basic fibroblast growth factors or VEGF 48 h prior to radiation prevents this acute microvascular loss and preserves long-term salivary flow [8]. These findings underscore endothelial cells as a primary target of radiation-induced damage. Furthermore, ionizing radiation has been shown to compromise vascular integrity by disrupting both TJs and AJs in microvascular endothelial cells, increasing endothelial barrier permeability [94,102]. Exposing murine microvascular endothelial cells to 10 Gy radiation for 24 h induces a significant reduction in the mRNA levels of TJs (including occludin, claudin-5, ZO-1, and PECAM-1) and AJs (including VE-cadherin, β-catenin, and γ-catenin), accelerating junctional deterioration [94]. Nevertheless, the specific effect of radiation on salivary endothelial cell junctions remains largely undefined.

Moreover, exposure to ionizing radiation leads to a significant depletion of pericyte populations in salivary glands. In mouse models receiving 15 Gy irradiation, the proportion of PDGFRβ-positive pericytes in the submandibular gland decreases from approximately 2.9% to around 1.0% four weeks post-irradiation [60]. This reduction coincides with vascular damage and decreased microvessel density. Temporal analysis of human salivary gland tissues reveals a similar rapid pericyte loss following irradiation. While little change is observed within the first 1–6 h, a significant decline becomes evident at approximately 24 h post-irradiation, continuing through 48 and 72 h [60]. Immunofluorescence analyses show that pericytes tightly enwrap CD31-positive blood vessels and are positioned near parasympathetic nerve fibers [60]. These findings suggest that pericytes are highly sensitive to radiation injury, and their simultaneous depletion alongside vascular density reduction strongly supports a mechanistic link between vascular damage and pericyte loss.

### 4.3. Diabetes Mellitus

Diabetes mellitus is a prevalent metabolic disorder that systemically affects multiple organs, including the salivary glands. Diabetic conditions impair both the morphology and function of salivary glands, typically leading to enlarged acini and secretory granules, atrophic ducts, and reduced salivary flow [95]. Hyperglycemia, a hallmark of diabetes, is a known driver of systemic endothelial dysfunction, increasing vascular permeability and impairing the expression of TJ proteins. For instance, reduced expression of claudin-5, occludin, and ZO-1 has been observed in the vascular endothelial cells of the kidney, retina, and brain under diabetic conditions, leading to disruption of endothelial barrier integrity [103,104,105]. Consistent with these in vivo findings, high glucose exposure decreases occludin and ZO-1 expression and increases permeability in cultured human umbilical vein endothelial cells [96].

In addition to systemic vasculature, hyperglycemia may also disrupt the microcirculation of salivary glands. In the submandibular glands of db/db mice, a spontaneous model of type 2 diabetes, TJ protein expression is significantly altered. Claudin-1 and -3 are upregulated, whereas claudin-4, occludin, and ZO-1 are significantly downregulated, suggesting that compromised TJ integrity may contribute to diabetes-induced hyposalivation [95]. Furthermore, proteome analysis of saliva samples from children and adolescents with diabetes have identified several differentially expressed proteins (e.g., PLG, SERPING1, SERPINC1, APOA2, FGB, and A2M) that are closely associated with endothelial dysfunction [106]. VEGF, a key regulator of vascular permeability, has also been implicated in diabetic complications. VEGF expression is elevated in the salivary glands of diabetic rat models, and administration of anti-VEGF antibodies can attenuate lymphocytic infiltration and significantly improve salivary secretory function [9,107,108]. Increased VEGF levels have also been detected in the saliva of pregnant women with diabetes [109]. While VEGF is known to induce occludin trafficking via phosphorylation and ubiquitination to increase retinal endothelial permeability [110,111], whether elevated VEGF similarly disrupts endothelial TJs and vascular permeability in diabetic salivary glands remains an important area for future investigation.

### 4.4. Vascular Barrier Disruption in Fibrotic Diseases

Salivary gland fibrosis is a common pathological outcome in various chronic inflammatory salivary gland disease. It is characterized by the persistent activation of fibroblasts and excessive deposition of extracellular matrix, which ultimately causes irreversible impairment of salivary gland function. Emerging evidence highlights that the disruption of the vascular endothelial barrier serves as a critical pathological event driving the progression of salivary gland fibrosis. In murine submandibular gland duct ligation models, fibrotic remodeling is accompanied by a marked increase in vascular permeability, demonstrated by enhanced leakage of fluorescent dextran tracers. This leakage occurs in parallel with the upregulation and mislocalization of the TJ protein claudin-5 from the apical membrane to the lateral and cytoplasmic compartments [10]. These structural alterations are closely linked to inflammatory signaling. Persistent inflammatory stimulation elevates the expression of monocyte chemoattractant protein-1 (MCP-1), which further disrupts endothelial barrier integrity and drives claudin-5 redistribution through activation of the ERK1/2 signaling pathway [10]. Similar claudin-5 alterations are observed in patients with chronic sialadenitis and IgG4-RS [10]. Notably, pharmacological intervention with the TJ modulator AT-1001 partially restores endothelial integrity, reduces vascular leakage, and significantly attenuates glandular fibrosis, underscoring a causal relationship between endothelial barrier disruption and fibrogenesis [10]. Therefore, reestablishing TJ integrity represents a promising therapeutic strategy for managing fibrotic salivary gland diseases. Finally, recent single-cell RNA sequencing of stromal-enriched cells from adult mouse submandibular and sublingual glands reveals that during glandular regeneration following deligation, endothelial interactions with pericytes, glial cells, fibroblasts, and immune cells are significantly enhanced, further highlighting the dynamic role of the vasculature in tissue repair [6].

### 4.5. Salivary Gland Tumors

Salivary gland tumors comprise a heterogeneous group of neoplasms with diverse histological subtypes, biological behaviors, and clinical outcomes. Growing evidence indicates that the disruption of the vascular endothelial barrier and alterations in cell–cell junction proteins play a pivotal role in the pathogenesis of salivary gland tumors. In salivary gland malignancies, particularly adenoid cystic carcinoma, a significant reduction or complete loss of junctional proteins is frequently observed [112,113,114,115]. For instance, the expression of E-cadherin is often downregulated in high-grade tumors, which correlates with increased invasiveness, lymph node metastasis, and poorer clinical prognosis [113,115]. Moreover, the catenin family shows a notable decrease in both benign and malignant salivary gland tumors, with the most significant loss reported in high-grade carcinomas [114,116,117]. The targeted ablation of p120-catenin in mouse salivary glands blocks acinar development and induces E-cadherin reduction, thereby promoting the formation of intraepithelial neoplasia and directly implicating p120-catenin loss in the early stages of tumorigenesis [118].

The tumor microenvironment also actively remodels the local vasculature, leading to a compromised endothelial barrier that is crucial for tumor progression. Endothelial cells within the tumor microenvironment often acquire an activated phenotype characterized by increased microvessel density, abnormal vessel morphology, and enhanced expression of angiogenic and inflammatory mediators, particularly VEGF-related pathways, which may weaken vascular junctional stability and facilitate plasma protein extravasation, stromal expansion, and tumor invasion [11,99,119,120]. Recent single-cell RNA sequencing analyses of salivary gland tumors have provided unprecedented resolution of the cellular heterogeneity within the tumor microenvironment, revealing intricate crosstalk between tumor cells, cancer-associated fibroblasts, immune cells, and the vasculature that collectively drive tumor progression [97,98,121]. Collectively, these findings underscore that targeting the restoration of junctional integrity or inhibiting the pathways that destabilize the vascular barrier may represent a promising therapeutic avenue for managing salivary gland tumors.

**Table 2:** Vascular endothelial barrier disruption in salivary gland diseases.

Disease Context	Key Endothelial Findings	Alterations in TJ/AJ Proteins	Supporting Models	Potential Therapeutic Implications
Sjögren’s syndrome (SS)	1. Increased microvessel diameter, blood flow, and vascular permeability 2. Excessive lymphocyte infiltration enabled by a compromised barrier	1. Claudin-5: Upregulated and redistributed from the membrane to the cytoplasm 2. Alterations are mediated by inflammatory cytokines like IFN-γ	1. Clinical: SS patients [101] 2. Animal: Non-obese diabetic mouse model [7] 3. In vitro: Human microvessel endothelial cells stimulated with IFN-γ [7]	1. Modulating claudin-5 expression or localization to restore endothelial barrier integrity and limit lymphocyte infiltration 2. Targeting IFN-γ signaling
IgG4-related sialadenitis (IgG4-RS)	1. Enhanced leukocyte transendothelial migration and obliterative phlebitis 2. Activation of ERK1/2 pathway in endothelial cells	1. Claudin-5: Upregulated and mislocalized from apicolateral membrane to lateral membrane and cytoplasm 2. Other TJs: Reduced mRNA expression of claudin-3, -4, -6, -7, -8, -10, -12, occludin, and ZO-1	1. Clinical: Submandibular gland tissues from IgG4-RS patients [10,93] 2. Proteomics: Analysis identified enrichment in leukocyte migration pathways [93]	1. Inhibiting the p-ERK1/2 signaling pathway 2. Modulating claudin-5 expression or localization to restore endothelial barrier integrity and limit lymphocyte infiltration
Radiation-induced injury	1. Acute and significant reduction in microvessel density 2. Depletion of pericyte populations, compromising vessel stability	1. TJ: Reduced mRNA expression of occludin, claudin-5, ZO-1, and PECAM-1 2. AJ: Reduced mRNA expression of VE-cadherin, β-catenin, and γ-catenin	1. Animal: Mice exposed to 15 Gy radiation [8,60] 2. In vitro: Murine microvascular endothelial cells exposed to 10 Gy radiation [94]	1. Strategies to stabilize TJs/AJs or protect pericytes 2. VEGF pretreatment
Diabetes mellitus	1. Systemic endothelial dysfunction 2. Increased vascular permeability 3. Elevated VEGF expression in diabetic salivary glands	1. Systemic: Reduced claudin-5, occludin, and ZO-1 in kidney, retina, and brain vasculature 2. Salivary gland: Upregulation of claudin-1, -3; significant downregulation of claudin-4, occludin, and ZO-1	1. Animal: db/db mouse model of type 2 diabetes [95]; diabetic rat models [107,108] 2. In vitro: High glucose exposure in HUVECs [96] 3. Clinical: Salivary proteomics analysis in diabetic children [106]	1. Targeting hyperglycemia-induced signaling pathways 2. Investigating the role of VEGF inhibition in preventing diabetes-associated hyposalivation and endothelial barrier disruption
Salivary gland fibrosis	1. Increased in vascular permeability 2. Persistent inflammatory stimulation drives barrier disruption	1. Claudin-5: Upregulated and mislocalized from apical to lateral/cytoplasmic compartments 2. Alterations are driven by the MCP-1/ERK1/2 pathway	1. Animal: Murine submandibular gland duct ligation model [10] 2. Clinical: Submandibular gland tissues from chronic sialadenitis and IgG4-RS patients [10]	1. Pharmacological modulation of TJs (e.g., AT-1001) to restore barrier integrity 2. Targeting the MCP-1/ERK1/2 signaling pathway
Salivary gland tumors	1. Increased microvessel density and abnormal morphology 2. Disrupted endothelial barrier creating a permissive gateway for tumor cell intravasation and metastasis	1. Epithelial junctions: Significant reduction/loss of E-cadherin and catenins 2. Endothelial junctions: VEGF pathway activation weakens vascular junctional stability	1. Clinical: Salivary gland tumor tissues [112,113,114,115] 2. Animal: Targeted ablation of p120-catenin in mouse salivary glands [118] 3. Omics: Single-cell RNA sequencing reveals complex crosstalk between tumor cells and the vasculature [97,98,121]	1. Restore junctional integrity and inhibit metastasis or improve drug delivery 2. Targeting pathways that destabilize the vascular barrier (e.g., VEGF signaling)

AJ, adherens junction; ERK, extracellular signal-regulated kinase; HUVEC, human umbilical vein endothelial cell; IFN-γ, interferon-γ; MCP-1, monocyte chemoattractant protein-1; PECAM-1, platelet endothelial cell adhesion molecule-1; TJ, tight junction; VEGF, vascular endothelial growth factor.

## 5. Therapeutic Strategies Targeting Endothelial Barrier

The endothelial barrier has been increasingly recognized as a critical and promising therapeutic target for multiple diseases. Early pharmacological approaches mainly relied on broad anti-inflammatory or anti-angiogenic interventions, such as glucocorticoids, anti-VEGF agents, and cytokine inhibitors, which indirectly ameliorate endothelial barrier dysfunction and vascular leakage by suppressing inflammation and angiogenesis [122]. In recent years, with the in-depth understanding of barrier biological mechanisms, drug development has gradually shifted from symptomatic treatment toward precise, mechanism-based modulation of endothelial/epithelial barrier function. The emerging therapeutic modalities, although explored primarily in non-salivary vascular systems, focus on directly or indirectly modulating TJs, AJs, pericyte–endothelial interactions, and intracellular signaling pathways that govern junctional assembly, actomyosin cytoskeletal contraction, and paracellular permeability.

Current endothelial/epithelial barrier-targeted therapeutic strategies can be divided into two major categories: barrier-protective and barrier-opening strategies (summarized in detail in Table 3). For inflammatory diseases characterized by pathological vascular hyperpermeability, such as sepsis, acute lung injury, and stoke-induced cerebral edema, the core therapeutic principle is to restore and stabilize endothelial barrier integrity [123,124]. This goal is achieved by upregulating the expression of core TJ proteins, stabilizing the interaction between TJs and AJs, and inhibiting intracellular signaling pathways that trigger junction disassembly and cytoskeletal contraction.

As a representative TJ regulator, larazotide acetate (also known as AT-1001), an octapeptide zonulin receptor antagonist, can effectively block the disassembly of the ZO-1 complex and prevent the subsequent excessive opening of the paracellular barrier. Accumulating preclinical studies have verified its potent efficacy in rescuing epithelial and endothelial barrier function in colitis, vasculitis, organ fibrosis, arthritis, and liver injury models [125]. Consistent with these findings, our previous work on salivary gland diseases further revealed that AT-1001 treatment significantly enhances endothelial barrier function and alleviates ligation-induced acinar atrophy and glandular fibrosis [10], providing initial, compelling evidence for the therapeutic potential of TJ modulators in salivary gland disorders. However, further investigation is required to delineate their endothelial cell-specific mechanisms, tissue selectivity, and long-term safety profile.

In addition to barrier-protective therapies, reversible and safe barrier-opening strategies have also been developed to address the poor tissue penetration and low bioavailability of high-molecular-weight drugs. A variety of TJ modulators and permeation enhancers have exhibited favorable effects in preclinical studies, which can effectively improve drug absorption in ocular, intranasal and intestinal tissues, and reverse the resistance of solid tumors to monoclonal antibody therapy and chemotherapy [126,127,128].

Despite these promising preclinical advances, the clinical translation of TJ modulators still faces considerable bottlenecks, including insufficient therapeutic efficacy, poor tissue specificity, irreversible barrier disruption, and potential safety risks. Non-specific systemic inhibition or opening of the endothelial barrier may lead to uncontrolled vascular leakage, widespread tissue edema, and even life-threatening systemic toxicity. To overcome these limitations, current research frontiers are committed to developing localized targeted drug delivery systems and utilizing the unique pathological microenvironment of diseased tissues to achieve precise site-specific drug activation and barrier regulation (e.g., tumor vasculature and ischemic penumbra). Future drug development should prioritize the screening and design of novel TJ modulators with high tissue specificity, well-defined molecular mechanisms, reversible regulatory effects, and reliable therapeutic safety windows. Applying these next-generation strategies to the salivary gland microvasculature, especially when combined with optimized delivery formulations, will be a critical step in advancing the translational potential for treating glandular disorders.

**Table 3:** Therapeutic agents targeting endothelial/epithelial junctions and junction-associated permeability mechanisms.

Therapeutic Strategy	Target Protein/ Pathway	Therapeutic Agent	Mechanism	Disease Context/ Indication	Developmental Status	Ref.
Close the barrier (to reduce leakage)	Zonulin/ZO-1	Larazotide acetate (AT-1001)	A zonulin receptor antagonist that prevents TJ disassembly, thus stabilizing the ZO-1 complex.	Celiac disease and systemic endothelial/ mucosal barrier dysfunction	Phase III clinical trials	[125]
	JAM-A	Anti-JAM-A antibodies	Blocks JAM-A-mediated leukocyte transmigration	Ischemia–reperfusion injury	early development	[129]
	JAM-C	Soluble JAM-C /Anti-JAM-C antibody	Prevents transmigration of leukocytes, reducing inflammation and edema.	Acute lung injury, sepsis, and arthritis	Preclinical	[130,131,132,133]
	VE-cadherin	Sitagliptin (DPP4 inhibitor)	Inhibits DPP4-mediated procalcitonin activation, preventing downstream VE-cadherin disassembly and vascular leakage	Sepsis and capillary leakage	Preclinical/ translational	[123]
	RhoA/ROCK–MLCK cytoskeletal axis	Fasudil, Y-27632	Inhibits ROCK, preventing actomyosin-driven cellular contraction and subsequent tearing of intercellular TJs/AJs	Sepsis, pulmonary edema, and inflammatory hyperpermeability	Clinically approved/ preclinical	[124,134,135]
	Endothelial-pericyte crosstalk	Imatinib (PDGFR-β inhibitor), recombinant Ang-1	Modulates pericyte coverage and prevents pathological VEGF-driven TJ disassembly	Alzheimer’s disease, diabetic retinopathy, and neurovascular injury	Preclinical	[136,137]
Open the barrier (to enhance drug delivery)	Claudins	C-CPE and derivatives	Binds directly to specific claudins to modulate TJ sealing and increase paracellular permeability	Drug delivery across claudin-expressing barriers	Preclinical/ experimental	[127,128,138]
	Claudin-5	C5C2 peptidomimetic	Binds extracellular loop of claudin-5, transiently disrupting BBB homophilic interactions	Neuro-drug delivery (CNS diseases)	Preclinical	[139]
	Claudin-5	siRNA/AAV-mediated shRNA	Suppresses *CLDN5* gene expression, leading to a temporary increase in BBB paracellular permeability.	CNS gene therapy, Alzheimer’s disease, and glioblastoma	Preclinical	[140,141,142,143]
	Occludin	Occludin extracellular loop peptides	Competitively inhibits occludin assembly, increasing macromolecular permeability.	Drug delivery across blood–testis barrier	Preclinical	[144,145,146]
	Zonulin/ZO-1	Larazotide acetate (AT-1002)	Increased permeability by reversible opening of TJs	Increase intestinal, intranasal, intratracheal, or transdermal penetration of various compounds	Clinical (Phase III for celiac)	[126,147]
	TJ/AJ protein interactions	HAV peptides; synthetic cadherin-interfering peptides	Competitively bind extracellular domains of junctional proteins to transiently increase paracellular space	CNS drug delivery	Preclinical	[148,149,150]

AJ, adherens junction; Ang-1, angiopoietin-1; BBB, blood–brain barrier; C-CPE, C-terminal fragment of *Clostridium perfringens* enterotoxin; CNS, central nervous system; DPP4, dipeptidyl-peptidase 4; HAV peptides, His-Ala-Val peptides; JAM, junctional adhesion molecule; PDGFR-β, platelet-derived growth factor receptor β; ROCK, Rho-associated coiled-coil kinase; TJ, tight junction.

## 6. Conclusions and Future Perspectives

The salivary gland vascular endothelial barrier plays a pivotal role in maintaining glandular homeostasis, intricately balancing rapid fluid fluxes during saliva secretion with the prevention of the excessive leakage of plasma proteins and immune cells. This function is largely governed by the structural integrity of endothelial TJs, among which claudin-5 has been identified as a key sealing component. However, the salivary endothelial barrier is not a static structure. Its permeability is dynamically regulated by multiple autonomic signaling pathways and undergoes structural remodeling in response to various physiological and pathological stimuli.

Despite these insights, significant knowledge gaps and methodological challenges persist. Important questions remain regarding the roles of additional junctional components, spatiotemporal interactions among TJs, AJs, and pericytes, as well as the potential influence of mechanical stimuli on endothelial barrier regulation. Current progress is also constrained by two major limitations: (1) the difficulty in distinguishing the contributions of the endothelial barrier from the adjacent epithelial barrier, which complicates the interpretation of salivary content changes; and (2) the scarcity of well-characterized salivary endothelial cell lines and robust in vitro models, which limits mechanistic studies. Addressing these limitations will enable a deeper mechanistic understanding and pave the way for novel barrier-targeted interventions for salivary gland disorders.

Growing evidence suggests that endothelial barrier dysfunction is not merely a secondary consequence of tissue injury but may act as an important pathogenic driver in multiple glandular disorders. Addressing this, our review highlights that targeting the endothelial barrier integrity is emerging as a promising therapeutic strategy for salivary gland diseases. Interventions aimed at correcting claudin-5 dysregulation, for instance, have shown encouraging preclinical potential in ameliorating TJ-related disorders. However, it is imperative to acknowledge that these approaches largely remain in the preclinical stage and are mechanistically incomplete. Bridging the translational gap requires developing reliable clinical assessment methods, such as non-invasive imaging and biomarker identification, and refining targeted drug delivery systems. Further in-depth research into the fundamental mechanisms of salivary endothelial barrier regulation will be essential to fully realize its diagnostic and therapeutic potential.

## Data Availability

No new data were created or analyzed in this study. Data sharing is not applicable to this article.
